# HINT2 protects against pressure overload‐induced cardiac remodelling through mitochondrial pathways

**DOI:** 10.1111/jcmm.18276

**Published:** 2024-03-28

**Authors:** Nan Zhang, Zi‐Ying Zhou, Yan‐Yan Meng, Hai‐Han Liao, Shan‐Qi Mou, Zheng Lin, Han Yan, Si Chen, Qi‐Zhu Tang

**Affiliations:** ^1^ Department of Cardiology Renmin Hospital of Wuhan University Wuhan China; ^2^ Hubei Key Laboratory of Metabolic and Chronic Diseases Wuhan China

**Keywords:** cardiac remodelling, HINT2, hypertrophy, mitochondrial complex I, NDUFs

## Abstract

Histidine triad nucleotide‐binding protein 2 (HINT2) is an enzyme found in mitochondria that functions as a nucleotide hydrolase and transferase. Prior studies have demonstrated that HINT2 plays a crucial role in ischemic heart disease, but its importance in cardiac remodelling remains unknown. Therefore, the current study intends to determine the role of HINT2 in cardiac remodelling. HINT2 expression levels were found to be lower in failing hearts and hypertrophy cardiomyocytes. The mice that overexpressed HINT2 exhibited reduced myocyte hypertrophy and cardiac dysfunction in response to stress. In contrast, the deficiency of HINT2 in the heart of mice resulted in a worsening hypertrophic phenotype. Further analysis indicated that upregulated genes were predominantly associated with the oxidative phosphorylation and mitochondrial complex I pathways in HINT2‐overexpressed mice after aortic banding (AB) treatment. This suggests that HINT2 increases the expression of NADH dehydrogenase (ubiquinone) flavoprotein (NDUF) genes. In cellular studies, rotenone was used to disrupt mitochondrial complex I, and the protective effect of HINT2 overexpression was nullified. Lastly, we predicted that thyroid hormone receptor beta might regulate HINT2 transcriptional activity. To conclusion, the current study showcased that HINT2 alleviates pressure overload‐induced cardiac remodelling by influencing the activity and assembly of mitochondrial complex I. Thus, targeting HINT2 could be a novel therapeutic strategy for reducing cardiac remodelling.

## 1 INTRODUCTION

Heart failure (HF) is a common advanced manifestation of various cardiovascular diseases that affects more than 64 million individuals worldwide.^1^ It is a significant clinical issue that poses a global public health threat. Myocardial remodelling is crucial in the development of HF and has been recognized as a major contributor to poor patient outcomes.^2^ While numerous interventions have been documented to reduce myocardial remodelling injury in animal models, none have clinically efficacy.^3^ Therefore, it is essential to thoroughly investigate the underlying mechanisms in order to find prospective therapeutic targets.

It is generally understood that energy delivery to the heart is crucial, and any interruption in cardiac energy supply can rapidly result in contractile dysfunction.^4^, ^5^ Previous studies have demonstrated that cardiac metabolic remodelling occurs early in the course of cardiac remodelling, even before indications of cardiac dysfunction emerge.^6^, ^7^ During hypertrophy and HF studies, the oxidative capacity of mitochondria was significantly reducted.^8^ The electron transport chain (ETC) consists of five multimeric complexes that facilitate redox reactions.^9^ The mitochondrial complex I (C‐I) is the most prevalent contributor to the pathophysiology of cardiovascular diseases.^10^ Hence, investigating the impact of targeting C‐I could be a compelling research topic in cardiac remodelling.

Histidine triad nucleotide‐binding protein 2 (HINT2), a member of the histidine triad protein superfamily, is predominantly located on the inner mitochondrial membrane and contains mitochondrial signal channel sequences.^11^ Deletion of HINT2 in mice impairs oxidative respiratory chain function and leads to significant acetylation of mitochondrial proteins, resembling mitochondrial damage observed in hypoxia.^12^, ^13^ In the heart, several studies have reported the significant role of HINT2 in ischemic heart disease.^11^, ^14^, ^15^ However, its effect on cardiac remodelling is unclear. This study aims to investigate HINT2's impact on cardiac remodelling and its mechanisms.

## 2 METHODS

### 2.1 Human heart samples

The source of the human heart samples was elucidated in a previous study.^16^ During cardiac transplantation, heart samples were specifically extracted from the left ventricles. Non‐failing controls were obtained from donors who had no previous cardiac disease. Informed consent was obtained from each donor, and the Renmin Hospital of Wuhan University Review Board approved all procedures and adhered to the Declaration of Helsinki.

### 2.2 Animals

After quarantine, male C57BL/6J mice were purchased from Beijing Huafukang Company and housed at the Hubei Provincial Model Animal Center. Mice with cardiomyocyte‐specific overexpression of HINT2 mice were generated. Mice carrying the B6‐TG (CAG‐HINT2) gene were obtained from Specialty Medicine Kang Biotechnology Company. Details about the gene's construction and identification are provided in Figure S1A–D. The B6‐CAG‐HINT2 mice were subsequently bred with B6‐Myh6Cre/+ mice, and the mice with B6‐Myh6Cre/+ −TG positive were identified and received an intraperitoneal injection of 5 mg/kg/d tamoxifen for 7 days to generate HINT2 overexpression mice.

Cardiomyocyte‐specific HINT2‐knockout (KO) mice were generated, as demonstrated in Figure S1E–G. The HINT2^fl/fl^ mice were crossed with alpha myosin heavy chain (αMHC)‐cyclization recombinase (Cre) mice. Tamoxifen (5 mg/kg/d) was then injected for 7 days to induce the depletion of HINT2 in cardiomyocytes. Wild‐type (WT) mice from the same litter were injected with an equivalent dose of tamoxifen as a control group for the HINT2‐KO mice.

All animal research conducted at Renmin Hospital of Wuhan University was authorized by the Animal Care and Use Committee. The studies were conducted in accordance with the guidelines for the Care and Use of Laboratory Animals provided by the United States National Institutes of Health (NIH Publication, revised 2011) (20181106).

### 2.3 Cardiac remoulding model

Following the administration of intraperitoneal sodium pentobarbital anaesthesia (50 mg/kg, once), male mice (8–10 weeks old, weighing 23.5–27.5 g) were randomly assigned to groups to undergo AB surgery, as previously described.^17^ After the induction of anaesthesia, the left thorax of each mouse was opened, and the thoracic aorta was carefully separated under a microscope. Subsequently, a 27‐gauge needle was positioned alongside the separated aortic segment, and the aorta was ligated by tying a slipknot around it using 7–0 silk sutures. Mice in the Sham group underwent a similar operation without aortic ligation.

### 2.4 Cardiac function

Following a 4‐week postoperative period, we conducted an examination of mouse heart echocardiography to measure left ventricular ejection fraction (LVEF), fraction shortening (LVFS) and LV end‐diastolic diameter (LVEDd), as outlined in our previous study.^18^ Additionally, LV pressure maximal rate of rise (dp/dt max) were recorded using a Millar Pressure‐Volume System (MPVS‐400; Millar Instruments). Subsequently, the mice were euthanized, and their heart weight to body weight (HW/BW) and heart weight to tibia length (HW/TL) were measured.

### 2.5 Histological analysis

The hearts were fixed using 10% neutral formalin, followed by dehydration and embedding in paraffin before being cut into 5 μm slices. Subsequently, the sections were subjected to haematoxylin–eosin staining (H&E), picrosirius red staining (PSR) and terminal deoxynucleotidyl transferaseerminal deoxynucleotidyl transferase‐mediated dUTP nick end labeling (TUNEL) staining as described in our previous study to assess the cross‐sectional area (CSA), volume of fibrosis in the heart, and cell death, respectively.^17^, ^19^ Additionally, cellular areas were calculated in more than 100 cells, the volume of fibrosis in the heart was measured in over 60 fields, the percentages of apoptotic cells were calculated by at least three independent individuals in a blinded manner. The data were then analysed using computerized analytic program (Image‐Pro Plus 6.0, Media Cybernetics, Bethesda, USA).

### 2.6 Isolation and treatment of neonatal rat cardiomyocytes (NRCMs)

NRCMs were isolated from the left ventricle using a previously established method.^20^ Subesquently, these cells were exposed to angiotensin II (Ang II, 1 μM) for 48 h to induce hypertrophy, while control cells were exposed to phosphate‐buffered saline (PBS).

Prior to Ang II treatment, NRCMs were pre‐incubated with adenoviruses containing either shHINT2 or shRNA for 24 h. Following 48 h of exposure to Ang II, the cells were collected for further research. Furthermore, NRCMs were infected with an adenovirus carrying the HINT2 gene (Ad‐HINT2) or a negative control (Ad‐GFP) for 4 h. To investigate the precise molecular mechanism underlying C‐I involvement in HINT2‐mediated cardiac hypertrophy, rotenone (Sigma‐Aldrich) was employed to inhibit C‐I in NRCMs.

### 2.7 RNA extraction and quantitative real‐time polymerase chain reaction (RT‐PCR)

The mRNA extraction and RT‐PCR were done as described in our previous study.^21^ In short, TRIzol reagent (Invitrogen, Carlsbad, CA, USA) was used to extract total RNA, while a Transcriptor First Strand cDNA Synthesis Kit (Roche, Basel, Switzerland) was utilized to produce cDNA from the extracted total RNA. Subsequently, SYBR Green I was used for quantitative RT‐PCR, and the primer sequences for HINT2, β‐actin, natriuretic peptides A (NPPA), natriuretic peptides B (NPPB), myosin‐6 (Myh6), myosin‐7 (Myh7), collagen‐3 (Col III), collagen‐1 (Col I), fibronectin (FN), connective tissue growth factor (CTGF), NADH dehydrogenase [ubiquinone] 1 alpha subcomplex subunit 13 (Ndufa13), NADH dehydrogenase [ubiquinone] 1 beta subcomplex subunit 11 (Ndufb11), Ndufb7, Ndufa2, Ndufa1, Ndufa3, NADH dehydrogenase [ubiquinone] iron–sulfur protein 7 (Ndufs7), Ndufb8, Ndufb6, Ndufa8 are provided in Table S1. The mRNA expression was standardized by comparing it to the levels of β‐actin.

### 2.8 Proteins extraction and western blotting assay

Proteins were extracted and a western blot analysis was conducted as previously described.^22^ Briefly, cardiac tissues, cultured cells or isolated mitochondria were subjected to RIPA lysis buffer or mitochondrial lysate. After BCA quantification, protein lysates were loaded into SDS‐PAGE gels for electrophoresis. The primary antibodies against the proteins HINT2 (ab128677), β‐actin (ab8227), CTGF (ab209780) and voltage‐dependent anion‐selective channel (VDAC, ab191440) were purchased from Abcam (Cambridge, UK). The proteins atrial natriuretic peptide (ANP, A22075), HINT2 (A24454), NDUFS7 (A24466), NDUFB8 (A19732), brain natriuretic peptide (BNP, A2179), beta myosin heavy chain (βMHC, A7564), COL1A1 (A1352) and FN (A12977) were purchased from ABclonal. Cell Signalling Technology supplied the secondary antibodies: anti‐rabbit (7074) and anti‐mouse (7076). The blots were detected using a chemiluminescence ECL kit (#1705062, Bio‐Rad, USA) and the band intensities were analysed using the Image Lab software.

### 2.9 Immunofluorescence staining

The immunofluorescence staining was conducted in accordance with manufacture's protocols. In summary, the cells on coverslips were fixed using 4% paraformaldehyde and permeabilized with 0.5% Triton X‐100. The cells were then subjected to overnight incubation at 4°C with α‐actinin (diluted 1:100). Finally, the cells were incubated with Alexa Fluor 488‐goat anti‐rabbit secondary antibody (1:200) for 1 h at room temperature. The nuclei were stained using DAPI (Sigma‐Aldrich) and the fluorescence images were captured using a specialized OLYMPUS DX51 fluorescence microscope (Tokyo, Japan). Subsequently, the data was analysed using Image‐Pro Plus 6.0.

### 2.10 Microarray performance and analysis

A microarray analysis was conducted on RNA samples obtained from the hearts of HINT2 transgenic (HINT2‐TG, *n* = 3) and non‐transgenic (NTG, *n* = 3) mice, 4 weeks after AB surgery. Cardiac tissue total RNA was extracted using TRIzol Reagent, followed by RNA integrity assessment using the Bioanalyzer 2100 system (Agilent Technologies, CA, USA). RNA‐seq libraries were then prepared, PCR products were purified using the AMPure XP system, and library quality was evaluated on the Agilent Bioanalyzer 2100 system. Clustering and sequencing were conducted on an Illumina Novaseq platform. The raw reads in fastq format were initially processed through in‐house perl scripts to obtain clean data, followed by mapping to the reference genome and prediction of novel transcripts using StringTie (v1.3.3b). The reads mapped to each gene were counted using featureCounts v1.5.0‐p3. Subsequently, the FPKM of each gene was calculated based on gene length and mapped reads count. Differential expression analysis between two groups was performed using the DESeq2 R package (1.20.0), with *p*‐values adjusted using the Benjamini and Hochberg's approach to control the false discovery rate (padj is its common form). Genes with an adjusted *p* < 0.05 found by DESeq2 were assigned as differentially expressed. The Kyoto Encyclopedia of Genes and Genomes (KEGG) and Gene Ontology (GO) enrichment analysis of differentially expressed genes (DEGs) were analysed using the cluster Profiler R package. The gene expression was normalized using a Z‐score normalization technique. The heat map and volcano plot were generated using the normalized expression of the DEGs. The raw data can be viewed in the GEO database (GSE260663).

### 2.11 Isolation of mitochondria and detecting mitochondrial membrane potential

After 4 weeks of operation, hearts from mice were rapidly excised and the left ventricular (LV) regions were separated. Mitochondria were isolated from the heart using the Mitochondria Isolation Kit for tissue (C3606, Beyotime) per manufacturer protocol. For NRCMs, after being treatment we used Mitochondria Isolation Kit for cell (C3601, Beyotime) according to the manufacturer protocol.

Freshly isolated mitochondria were used to measure mitochondrial membrane potential (MMP) (Δψm) by enhanced mitochondrial membrane potential assay kit with JC‐1 (C2003S, Beyotime) following the manufacturer's guidelines. Briefly, mixed the mitochondria with JC‐1 working solution and detected with fluorescence spectrophotometer. Time scan is performed directly with fluorescence spectrophotometer, excitation wavelength is 485 nm, emission wavelength is 590 nm.

In addition, excess mitochondria were cryopreserved in mitochondrial storage buffer for the detection of mitochondrial proteins and other related experiments.

### 2.12 Mitochondrial ETC complex I activity

The activity of mitochondrial complex I was assessed according to the manufacturer's protocol (ab109721, Abcam). In brief, resuspend the frozen mitochondria, determine protein concentration using the BCA method, then adjust the remaining sample to 5.5 mg/mL. After extraction, dilute samples in incubation solution to the desired concentration and load onto a plate. After incubation and washing, 200 μL of assay solution was added to each well. At room temperature, set the wavelength to 450 nm, and measure every minute for a total of 30 min. Shake well before each measurement. Measure OD values in kinetic mode, record the values, and calculate the rate/slope from 0 to 30 min.

### 2.13 Seahorse extracellular flux analyser assays

NRCMs were transfected with adenviruse to overexpress HINT2 or to KO HINT2 and the oxygen consumption rate (OCR) was determined following exposure to Ang II for 48 h. Seahorse XFe24 analyses were used to assess the OCR and adjust it to protein concentrations as previous described.^23^ In order to quantify ATP‐linked OCR, the basal cellular respiration was measured after an injection of the ATP synthase inhibitor oligomycin (1 μM). Maximal respiration was calculated using an uncoupling agent (FCCP 1 μM). Rotenone (1 μM) and Antimycin A (2 μM) were given to block complex I and III, respectively, and to assess non‐mitochondrial respiration.

### 2.14 Measuring ATP, NAD and NADH levels

Using the Enhanced ATP Assay Kit (S0027, Beyotime), the ATP levels were been detected. Utilizing the extracted mitochondria from the heart tissues or NRCMs and the NAD+/NADH Assay Kit with WST‐8 (S0175, Beyotime), the NAD and NADH levels were been measured. The protocol was carried out as directed by the kit.

### 2.15 Downloading and analysis of public datasets

We obtained the microarray of GSE36074,^24^ GSE198926^25^ and GSE42955^26^ from the National Center for Biotechnology Information (NCBI) GEO database. GSE36074 includes 14 mice from AB group and five mice from sham group. GSE42955 includes 12 human dilated cardiomyopathy (DCM) hearts and five normal hearts. GSE198926 includes four mice from transverse aortic constriction (TAC) group and four mice from sham group. The expression of HINT2 and the correlation of HINT2 with thyroid hormone receptor beta (THRB) were analysed by R package.

### 2.16 STATISTICAL ANALYSIS

The data were expressed as the Mean ± Standard error of the mean (SEM). The analysis was performed using GraphPad Prism software Version 8. For two groups, data was compared by the two‐tailed unpaired Student's *t*‐test; for more than two groups, data were analysed by one‐way ANOVA with Tukey post hoc analysis. *p* < 0.05 was considered statistically significant.

## 3 RESULTS

### 3.1 HINT2 expression is markedly downregulated in hypertrophic hearts

**FIGURE 1:**
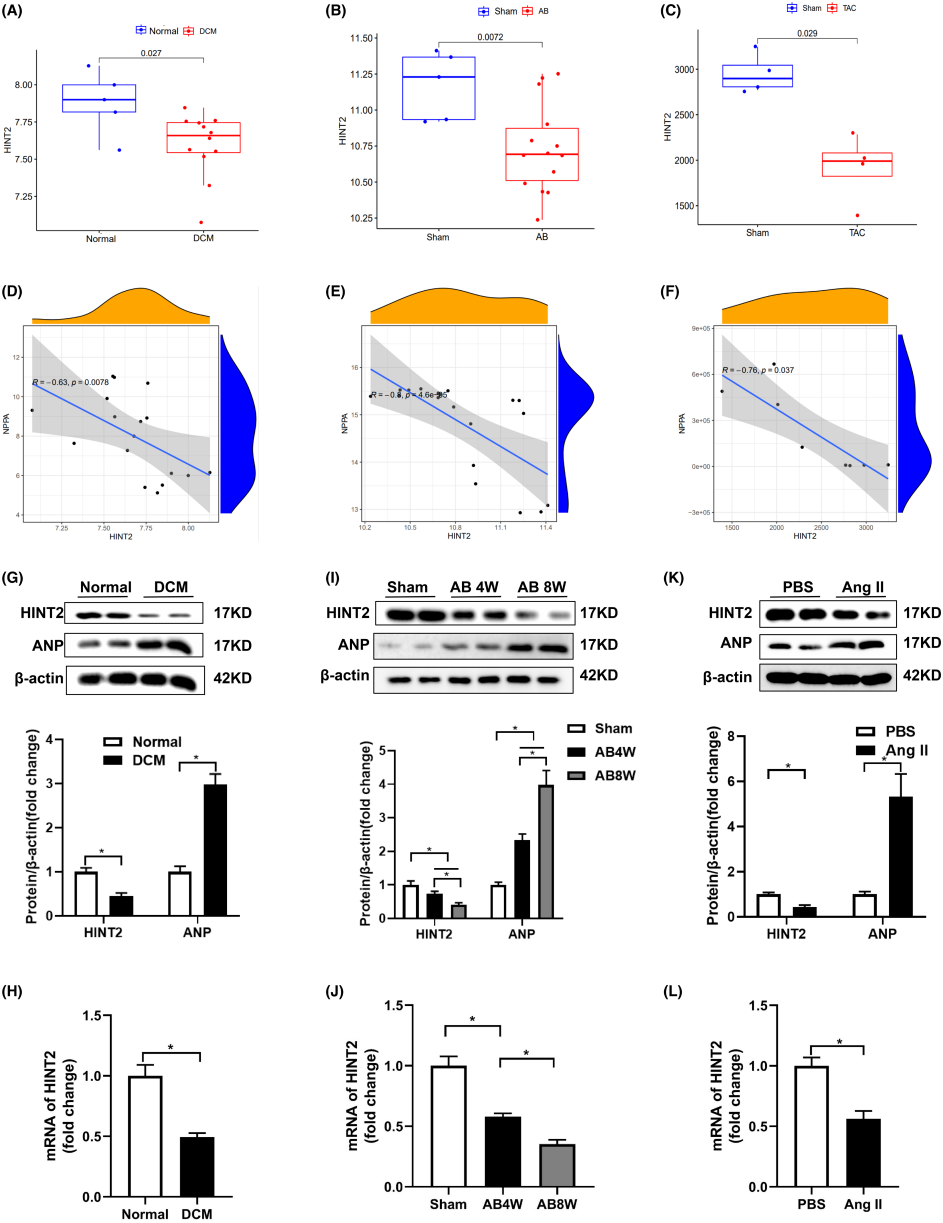
Histidine triad nucleotide‐binding protein 2 (HINT2) expression is markedly downregulated in hypertrophic hearts and might be a promising factor related to heart failure. (A–C) The expression of HINT2 in human dilated cardiomyopathy (DCM) hearts, mice with AB hearts and mice with transverse aortic constriction (TAC) hearts were significantly lower than that in normal hearts or sham hearts (A. GSE42955, B. GSE36074 and C. GSE198926). (D–F) The expression of HINT2 was significantly negatively correlated with the expression of natriuretic peptides A (NPPA), (A. GSE42955, B. GSE36074 and C. GSE198926). (G) Representative immunoblotting images of HINT2 and ANP protein levels (upper) in human heart tissues collected from DCM and normal donors and statistical analyses of HINT2 and ANP (blow), *n* = 6, *p < 0.05. (H) Relative mRNA levels of HINT2 in human heart tissue samples from DCM and normal donors, *n* = 6, *p < 0.05. (I) Representative immunoblotting images of HINT2 and ANP protein levels (upper) in heart tissues collected from sham, AB4W and AB8W mice and statistical analyses of HINT2 and ANP (blow), *n* = 6, *p < 0.05. (J) Relative mRNA levels of HINT2 in heart tissue samples from sham, AB4W and AB8W mice, *n* = 6, *p < 0.05. (K) Representative immunoblotting images of HINT2 and ANP protein levels (upper) in NRCMs exposed to Ang II or phosphate‐buffered saline (PBS) and statistical analyses of HINT2 and ANP (blow), *n* = 6, *p < 0.05. (L) Relative mRNA levels of HINT2 in NRCMs exposed to Ang II or PBS, *n* = 6, *p < 0.05.

First, we examined the data obtained from human DCM heart tissues and mice hypertrophic hearts, which were acquired from the GEO database (GSE42955 is shown in Figure 1A,D, GSE36074 is shown in Figure 1B,E and GSE198926 is shown in Figure 1C,F). Figure 1A–C demonstrates a notable decrease in HINT2 expression in human DCM heart tissues and mice AB or TAC hearts compared to normal hearts. Additionally, there is a notable negative correlation exists between the expression of HINT2 and NPPA (Figure 1D–F).

To validate these findings, we assessed HINT2 expression in cardiac samples from DCM patients and healthy donors. Increased levels of ANP indicated failing hearts. We observed a significant reduction in HINT2 levels in DCM hearts compared to normal hearts (Figure 1G), along with notable decreases in HINT2 mRNA levels in DCM hearts (Figure 1H). Additionally, significant reductions in both HINT2 protein and mRNA levels were observed in the cardiac tissues of mice at 4 and 8 weeks following AB surgery (Figure 1I,J). Incubation of NRCMs with Ang II for 48 h resulted in a substantial decrease in both the protein and mRNA levels of HINT2 compared to PBS‐treated cells (Figure 1K,L). These findings strongly suggest that HINT2 may be involved in cardiac remodelling.

### 3.2 HINT2 overexpression alleviated AB‐induced cardiac remodelling in vivo

**FIGURE 2:**
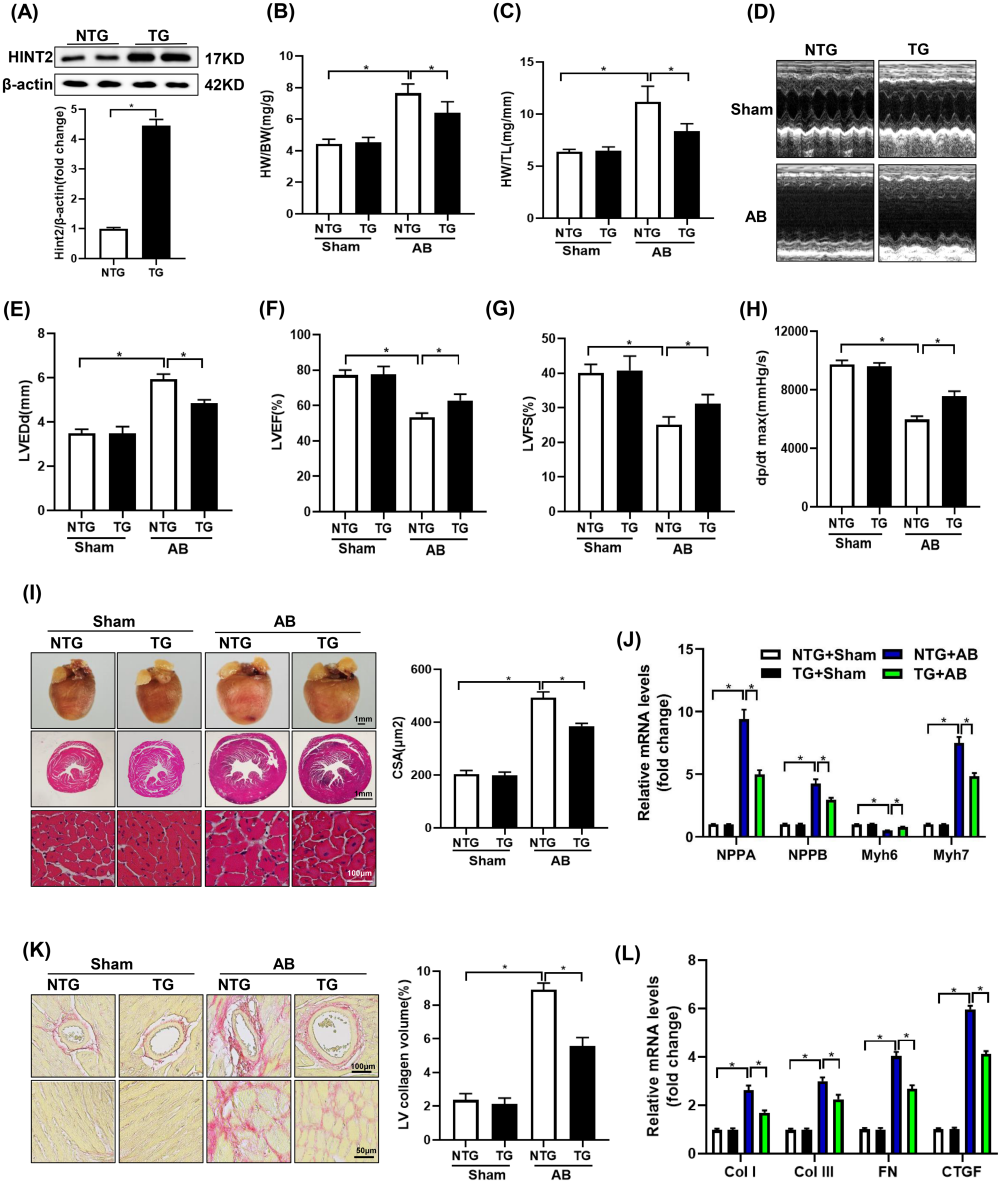
Cardiac specific overexpression of histidine triad nucleotide‐binding protein 2 (HINT2) attenuated AB‐induced cardiac remodelling in vivo. (A) Representative western blots (upper) and quantitative results (blow) of HINT2 expression in NTG and TG mice (*n* = 6, *p < 0.05). (B, C) Comparison of heart weight to body weight ratio (HW/BW), heart weight to tibia length ratio (HW/TL) between NTG and TG mice at 4 weeks after sham or AB surgery (*n* = 12, *p < 0.05). (D) Representative M echocardiographic pictures. (E–H) Echocardiographic and pressure volume loop assessments of LVEDd, LVEF, LVFS and dp/dt max between the indicated groups (*n* = 12, *p < 0.05). (I) Left, representative images of gross hearts, HE staining of heart sections in the indicated groups (*n* = 3). Right, morphometric results for the cross‐sectional area (CSA) of cardiomyocytes in the indicated groups (*n* ≥ 100 cells per group, *p < 0.05). (J) mRNA levels of the hypertrophic markers in each group (*n* = 6, *p < 0.05). (K) Left, representative images of PSR staining at 4 weeks after sham or AB surgery (*n* = 3). Right, quantitative results pertaining to LV collagen volumes in the indicated groups (*n* ≥ 40 fields per group, *p < 0.05). (L) Transcription levels of the fibrotic marker Col I, Col III, FN and CTGF in each group (*n* = 6, *p < 0.05). LVEDd, LV end‐diastolic diameter; LVEF, left ventricular ejection fraction; LVFS, left ventricular fraction shortening; PSR, picrosirius red staining.

To evaluate HINT2 overexpression, immunoblot assays were conducted. Figure 2A shows a notable increase in HINT2 protein expression in TG mouse hearts compared to NTG mice. In AB group, TG mice exhibited a significant decrease in cardiac enlargement compared to NTG mice, as indicated by reduced normalized HW/BW and HW/TL (Figure 2B,C).

The echocardiographic analysis of cardiac function revealed that HINT2 overexpression attenuated AB‐induced cardiac dysfunction. This was demonstrated by an increase in the LVEF and LVFS, in addition to the increase in dp/dt max and a decrease in LVEDd (Figure 2D–H).

TG mice showed reduced cardiac remodelling post‐AB compared to NTG mice, evidenced by decreased CSA (Figure 2I), less LV collagen deposition (Figure 2K), and decreased expression of hypertrophic markers like ANP, BNP, and βMHC, along with increased αMHC expression (Figure 2J, Figure S2A,B). Additionally, TG mice exhibited notably reduced expression of fibrosis markers like Col I, Col III, FN, and CTGF compared to NTG mice following AB surgery (Figure 2L, Figure S2A,B).

Furthermore, TUNEL was conducted to evaluate AB‐induced cell apoptosis. A notably elevated percentage of TUNEL‐positive nuclei was detected in the AB group. Conversely, HINT2 overexpression led to a significant reduction in the percentage of TUNEL‐positive cells induced by pressure overload (Figure S2C,D). Collectively, these findings suggest that the overexpression of HINT2 confers cardiac resilience against pressure overload‐induced stress by suppressing hypertrophy and fibrosis, decreasing cardiomyocyte apoptosis.

### 3.3 HINT2 knockout potentiates AB‐induced cardiac remodelling in vivo

**FIGURE 3:**
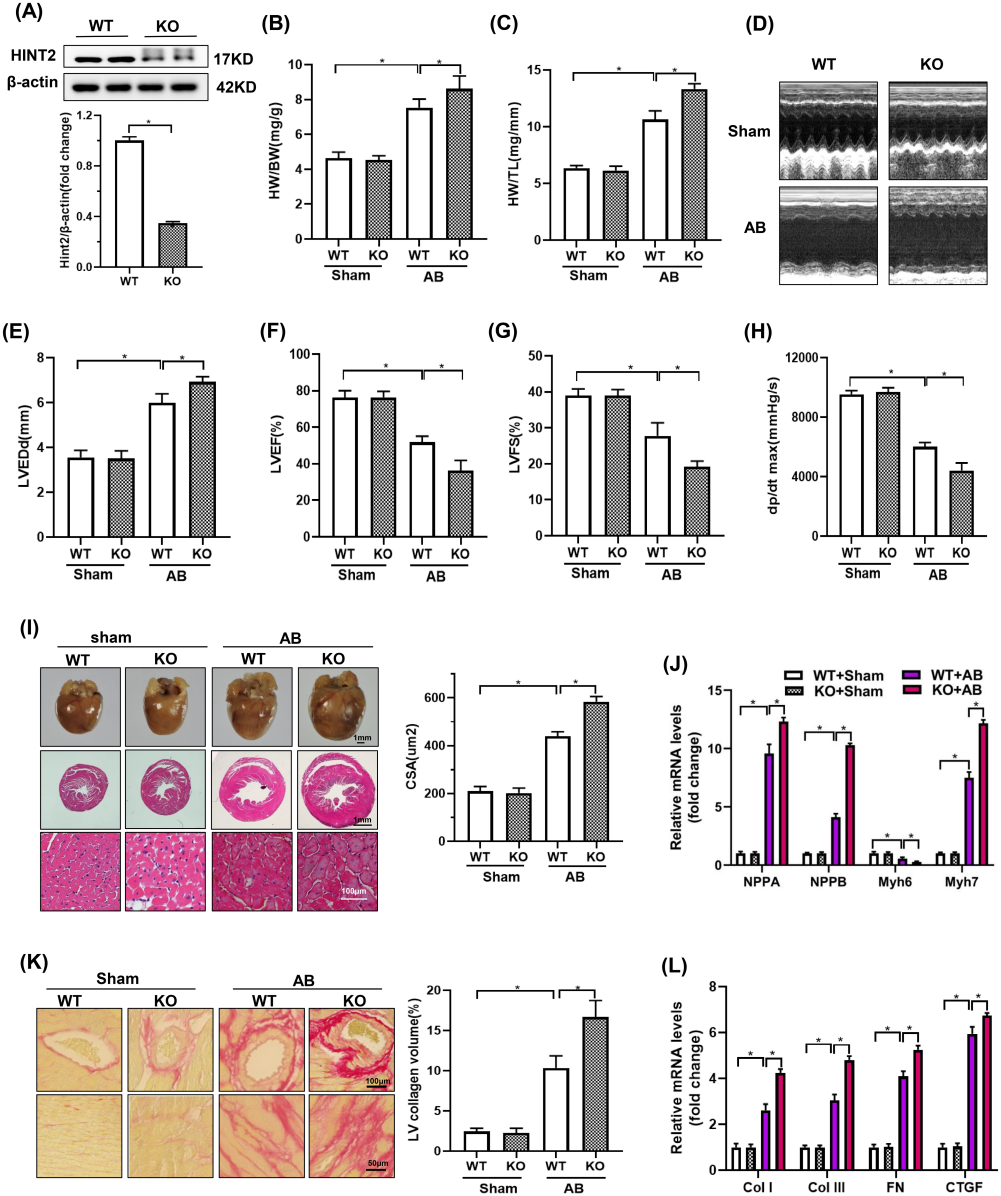
Histidine triad nucleotide‐binding protein 2 (HINT2) knockdown exacerbated AB‐induced cardiac remodelling and dysfunction. (A) HINT2 protein expression in the hearts from WT mice and KO mice (*n* = 6, *p < 0.05). (B, C) The ratio of HW/BW and HW/TL in WT and KO mice at 4 weeks after sham or AB surgery (*n* = 12, *p < 0.05). (D) Representative M echocardiographic images. (E–H) Echocardiographic and pressure volume loop assessments of LVEF, LVFS, LVEDd and dp/dt max in the indicated groups (*n* = 12, *p < 0.05). (I) Left, representative images of gross hearts, HE staining of heart sections in the indicated groups (*n* = 3). Right, quantification of the average cross‐sectional area (CSA) of cardiomyocytes in the indicated groups (*n* ≥ 100 cells per group, *p < 0.05). (J) mRNA levels of hypertrophic markers in the indicated mouse hearts (*n* = 6, *p < 0.05). (K) Left, representative images of cardiac PSR staining of the perivascular area (top; scale bar, 100 μm) and interstitial area (bottom; scale bar, 50 μm) at 4 weeks after sham or AB surgery (*n* = 6). Right, quantification of the LV collagen volume (*n* ≥ 40 fields per group, *p < 0.05). (L) Cardiac mRNA levels of fibrotic markers Col I, Col III, FN and CTGF in the indicated groups (*n* = 6, *p < 0.05). LVEDd, LV end‐diastolic diameter; LVEF, left ventricular ejection fraction; LVFS, left ventricular fraction shortening; PSR, picrosirius red staining.

Western blotting confirmed cardiac‐specific HINT2 KO, showing reduced HINT2 protein levels in the hearts of KO mice compared to WT mice (Figure 3A). While baseline heart phenotypes were unaffected by HINT2 deletion, it significantly exacerbated AB‐induced cardiac hypertrophy, evidenced by increased normalized HW/BW and HW/TL (Figure 3B,C). Furthermore, cardiac function analysis revealed that HINT2 deficiency worsened AB‐induced cardiac dysfunction, leading to decreased LVEF, LVFS, dp/dtmax, and increased LVEDd (Figure 3D–H).

H&E staining showed more severe AB‐induced pathological cardiac hypertrophy in HINT2‐KO mice compared to WT mice, characterized by enlarged cardiomyocyte CSA and increased levels of hypertrophic markers in HINT2‐KO mice (Figure 3I,J, Figure S2E,F). Additionally, PSR staining revealed more severe cardiac fibrosis in HINT2‐KO mice compared to WT mice in the AB group (Figure 3K), supported by increased expression of fibrotic markers (Figure 3L, Figure S2E,F). Additionally, a TUNEL assay was conducted to evaluate cell apoptosis. A significantly higher percentage of TUNEL‐positive nuclei was observed in the AB group. HINT2 deficiency led to a significant increase in the percentage of TUNEL‐positive cells (Figure S2G,H).

Taken together, these findings indicate that HINT2 absence exacerbates cardiac injury induced by increased cardiac pressure, amplifying both cardiac hypertrophy and fibrosis, aggravated AB‐induced cardiomyocyte apoptosis.

### 3.4 HINT2 promotes ETC complex I and respiratory function

**FIGURE 4:**
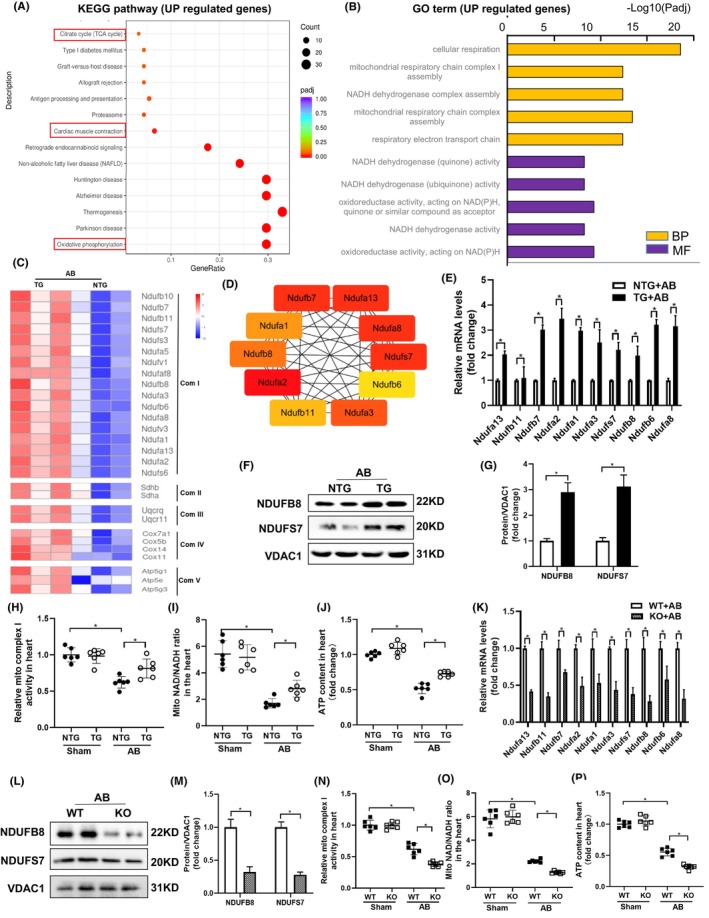
Histidine triad nucleotide‐binding protein 2 (HINT2) promotes ETC complex I and respiratory function. (A) Kyoto Encyclopedia of Genes and Genomes (KEGG) pathway enrichment analysis of the upregulated genes. (B) Gene Ontology (GO) term of the upregulated genes. (C) Heat map demonstrating mean normalized expression of transcripts encoding the subunits of the mitochondrial respiratory chain. RNA‐sequencing data from NTG and TG mouse heart 4 weeks post‐AB, *n* = 3/group. Red indicated high expression, and blue indicates low expression. (D) Protein–protein interaction network showed the top 10 genes. (E) Relative mRNA expression of ETC complex I genes in the heart from NTG and TG mice 4 weeks post‐AB. Data are expressed as the fold change from NTG mice 4 weeks post‐AB (*n* = 6, *p < 0.05). (F, G) Representative western blots (F) and quantitative results (G) of NDUFB8 and NDUFS7 expression in NTG and TG mice after AB4W (*n* = 4, *p < 0.05). (H–J) Activity of ETC complexes I in cardiac mitochondria (H), the NAD+/NADH ratio (I) and ATP content in heart isolated from NTG and TG mice 4 week after sham or AB operation (J) (*n* = 6, *p < 0.05). (K) Relative mRNA expression of ETC complex I genes in the heart from KO and WT mice 4 weeks post‐AB. Data are expressed as the fold change from WT mice 4 weeks post‐AB (*n* = 6, *p < 0.05). (L, M) Representative western blots (L) and quantitative results (M) of NDUFB8 and NDUFS7 expression in KO and WT mice after AB4W (*n* = 4, *p < 0.05). (N–P) Activity of ETC complexes I in cardiac mitochondria (N), the NAD+/NADH ratio (O) and ATP content (P) in heart isolated from KO and WT mice 4 week after sham or AB operation (*n* = 6, *p < 0.05).

To understand the protective effects of HINT2, we performed RNA‐seq assays on TG mouse hearts and NTG hearts after AB treatment. We identified 225 upregulated and 351 downregulated genes in TG mice compared to NTG mice after AB treatment (Figure S3A), the criteria is |log2Fold change| >0.5 and padj <0.05. KEGG pathway analyses showed that the upregulated genes were enriched in oxidative phosphorylation, cardiac muscle contraction, and citrate cycle pathway (Figure 4A), while downregulated genes were enriched in ECM‐receptor interaction, malaria, and focal adhesion pathway (Figure S4B). GO analyses of upregulated genes indicated prominent processes related to cellular respiration and assembly of mitochondrial respiratory chain complexe I (Figure 4B), with molecular functions associated with NADH activities. Conversely, downregulated genes were associated with extracellular matrix organization and collagen fibril organization pathway (Figure S4C). A significant proportion of upregulated genes were related to mitochondrial ETC complex I subunits (Figure 4C), with the top 10 hub genes belonging to complex I subunits (Figure 4D).

Transcriptomic results were confirmed by increased expression of NDUFs genes in TG mice compared to NTG mice 4 weeks after AB (Figure 4E). Evaluation of protein expression of mitochondrial ETC complex I confirmed that HINT2 overexpression led to increased expression of ETC complex I related proteins in AB treated group (Figure 4F,G). At 4 weeks post‐AB, TG mice showed increased activity of ETC complex I in cardiac mitochondria compared to NTG mice (Figure 4H). Accordingly, TG mice exhibited increased cardiac ATP biosynthesis and NAD+/NADH ratio compared to NTG mice 4 weeks after AB (Figure 4I,J).

To further confirm HINT2's significance in mitochondrial complex I, we investigated hearts from HINT2 KO mice after AB surgery. Results showed a notable reduction in transcriptional and protein levels of genes associated with mitochondrial complex I compared to WT mice (Figure 4K–M), along with a decline in mitochondrial function (Figure 4N–P). These findings collectively indicate that HINT2 stimulates ETC complex I activity, crucial for sustaining respiratory function, ATP generation, and balancing NAD^+^/NADH levels, essential for meeting cardiac energy demands under stress.

### 3.5 HINT2 protects against Ang‐II induced cardiomyocyte hypertrophy in vitro

**FIGURE 5:**
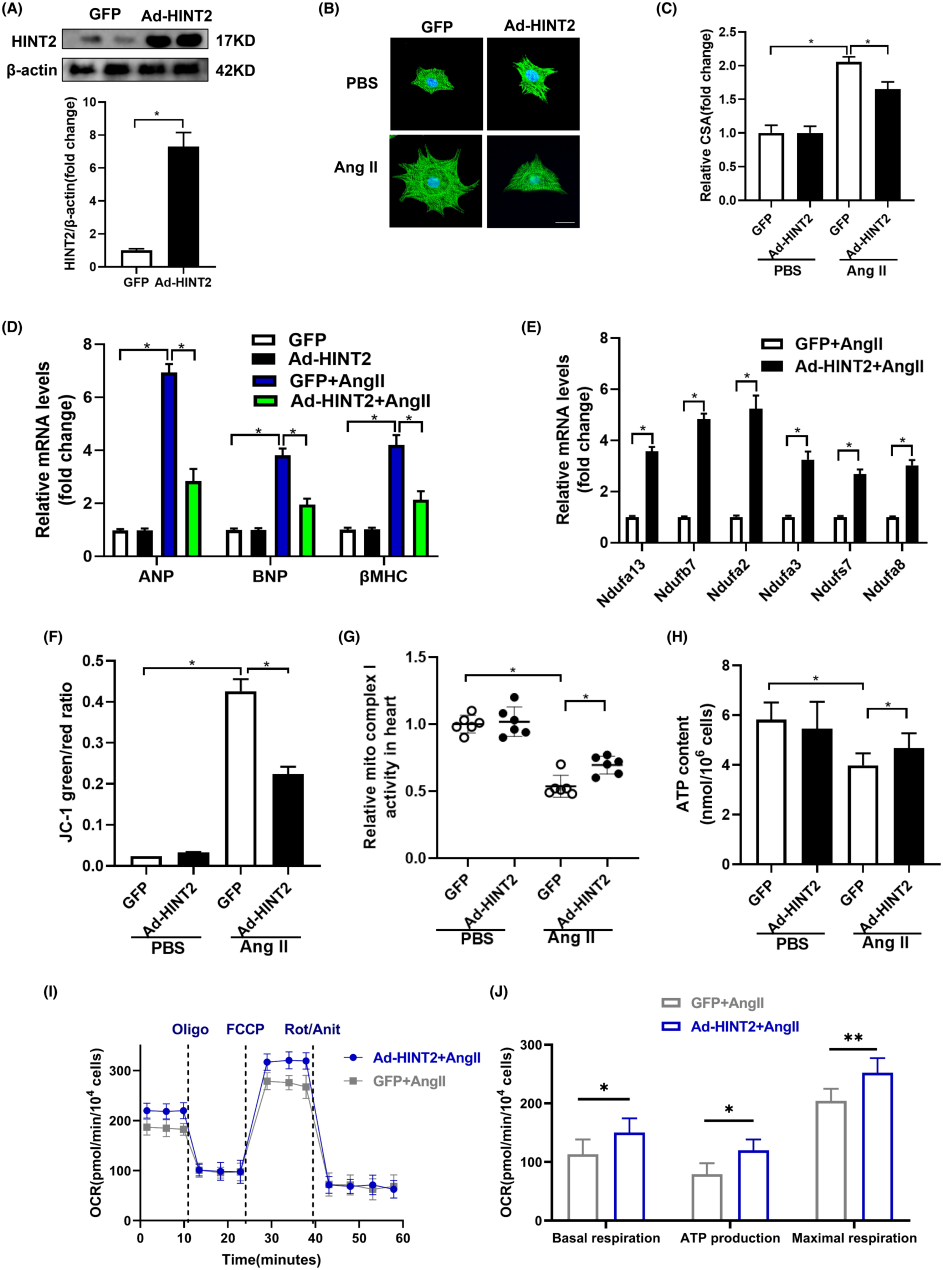
Histidine triad nucleotide‐binding protein 2 (HINT2) protects against Ang‐II induced cardiomyocyte hypertrophy in vitro. (A) Immunoblots (upper) and quantification (blow) of HINT2 protein expression after NRCMs were transfected with Ad‐GFP or Ad‐HINT2 (*n* = 6, *p < 0.05). (B, C) Representative images of α‐actin and DAPI stained NRCMs infected with GFP or Ad‐HINT2 before or being treated with phosphate‐buffered saline (PBS) or Ang II for 48 h. Scale bar, 20 μm (B). Quantification of the average cross‐sectional area (CSA) in the indicated group (C, *n* ≥ 50 cells per group, *p < 0.05). (D). mRNA levels of hypertrophic markers in the indicated groups (*n* = 6, *p < 0.05). (E) Relative mRNA expression of ETC complex I genes (*n* = 6, *p < 0.05). (F) JC‐1 detection of mitochondrial membrane potential (Δψm) (*n* = 6, *p < 0.05). (G) Activity of ETC complexes I in cardiac mitochondria in NRCMs infected with GFP or Ad‐HINT2 and treated with PBS or Ang II for 48 h (*n* = 6, *p < 0.05). (H) The content of ATP (*n* = 6, *p < 0.05). (I) In assays of mitochondrial respiration, maximum oxygen consumption rate (OCR) was measured in NRCMs cultured under Ang II or PBS for 48 h (*n* = 4, *p < 0.05). (J) Basal respiration; ATP production; Maximal respiration (*n* = 4, *p < 0.05).

We confirmed our findings through in vitro experimentation using isolated NRCMs, modulating HINT2 expression via adenovirus infection. Immunoblot assays confirmed successful HINT2 overexpression at the protein level in NRCMs (Figure 5A). Consistent with our in vivo observations, HINT2 overexpression attenuated Ang II‐induced cardiomyocyte hypertrophy, evidenced by decreased CSA (Figure 5B,C) and the mRNA and protein expression of hypertrophic markers ANP, BNP and βMHC (Figure 5D, Figure S4A,B).

Additionally, upregulation of genes associated with mitochondrial complex I was observed at the transcriptional level (Figure 5E). Preservation of Δψm was also noted in HINT2‐overexpressing NRCMs treated with Ang II, demonstrated by JC‐1 detection (Figure 5F). Moreover, HINT2 overexpression significantly impacted mitochondrial complex I activity in NRCMs exposed to Ang II (Figure 5G). ATP detection assay further revealed enhanced ATP synthesis in HINT2‐overexpressing NRCMs following Ang II treatment (Figure 5H).

We evaluated the impact of HINT2 on mitochondrial respiratory capacity by measuring the OCR (Figure 5I,J). HINT2 stimulation increased maximum OCR in NRCMs exposed to Ang II, suggesting a protective effect on mitochondrial respiration. HINT2 overexpression in NRCMs elevated basal, ATP‐linked, and maximum OCRs after Ang II treatment compared to GFP‐transfected cells. These findings demonstrate that HINT2 overexpression reduces cardiac hypertrophy‐related cell size by preserving mitochondrial respiration, ATP synthesis and MMP integrity, highlighting its crucial role in cellular well‐being during hypertrophic stress.

### 3.6 HINT2 deficiency aggravates Ang‐II induced cardiomyocyte hypertrophy in vitro

**FIGURE 6:**
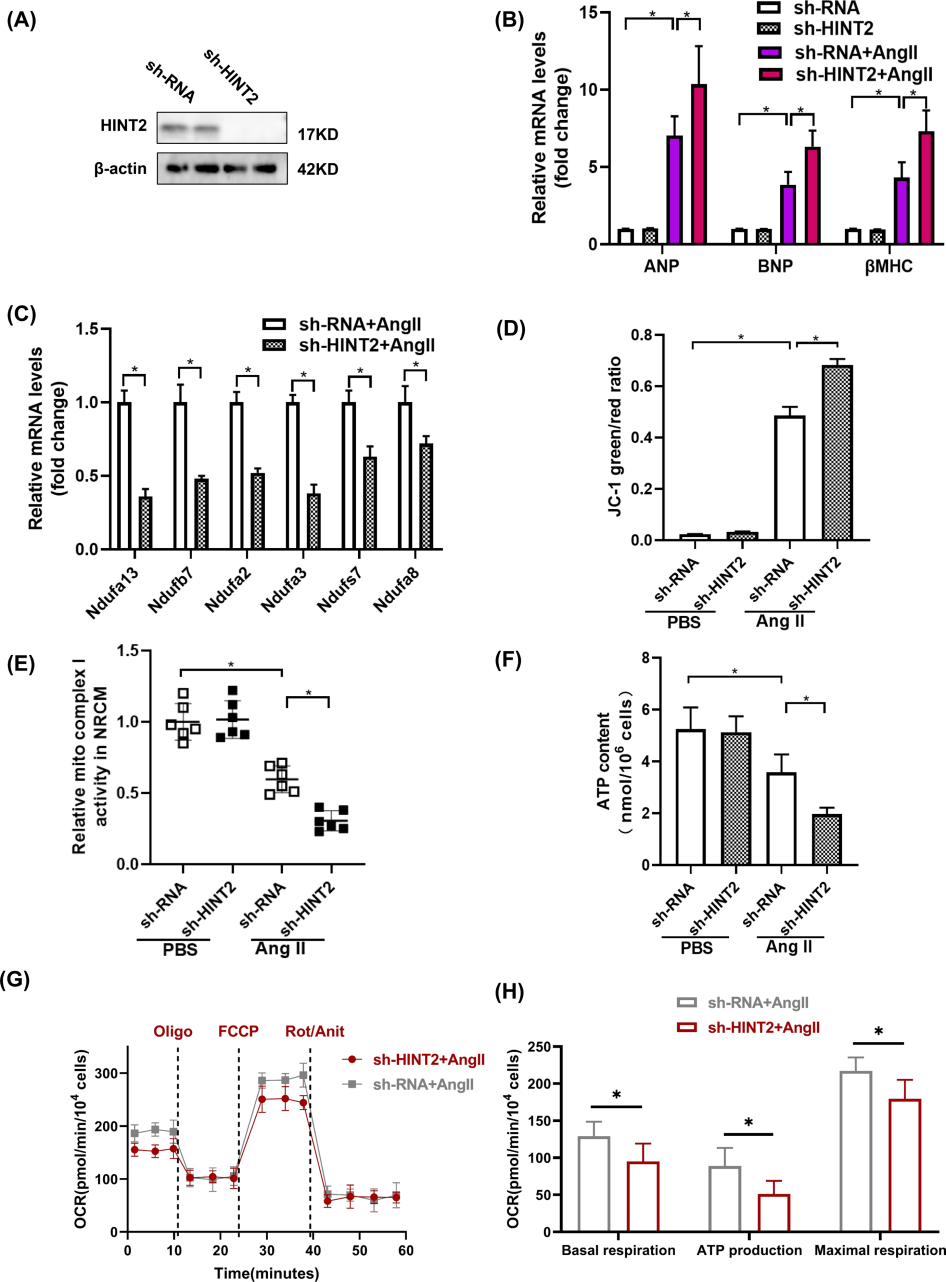
Histidine triad nucleotide‐binding protein 2 (HINT2) deficiency aggravates Ang‐II induced cardiomyocyte hypertrophy in vitro. (A) Immunoblots of HINT2 protein expression after NRCMs were transfected with sh‐RNA or sh‐HINT2 (*n* = 6, *p < 0.05). (B) mRNA levels of hypertrophic markers in the indicated groups (*n* = 6, *p < 0.05). (C) Relative mRNA expression of ETC complex I genes (*n* = 6, *p < 0.05). (D) JC‐1 detection of mitochondrial membrane potential (Δψm) (*n* = 6, *p < 0.05). (E) Activity of ETC complexes I in cardiac mitochondria in NRCMs infected with sh‐RNA or sh‐HINT2 and treated with phosphate‐buffered saline (PBS) or Ang II for 48 h (*n* = 6, *p < 0.05). (F) The production of ATP (*n* = 6, *p < 0.05). (G) Maximum oxygen consumption rate (OCR) was measured in NRCMs transfected with sh‐RNA or sh‐HINT2 and cultured under Ang II or PBS for 48 h (*n* = 4, *p < 0.05). (H) Basal respiration; ATP production; Maximal respiration (*n* = 4, *p < 0.05).

To validate our findings from HINT2‐overexpressing cells, we constructed an adenovirus to suppress HINT2 expression and introduced it into NRCMs (Figure 6A). Contrary to previous results, HINT2 knockdown resulted in increased hypertrophic markers with Ang II treatment (Figure 6B, Figure S4C,D), indicating worsened myocardial hypertrophy. Additionally, subunits associated with mitochondrial complex I showed downregulated transcriptional activity (Figure 6C). JC‐1 assay revealed decreased preservation of MMP (Figure 6D), reduced mitochondrial complex I activity (Figure 6E), decreased ATP content (Figure 6F), and weakened mitochondrial respiration capacity indicated by OCR (Figure 6G,H). These findings collectively demonstrate that HINT2 deficiency impairs mitochondrial function.

### 3.7 Mitochondrial complex I inhibition offsets the protective effect of HINT2 in vitro

**FIGURE 7:**
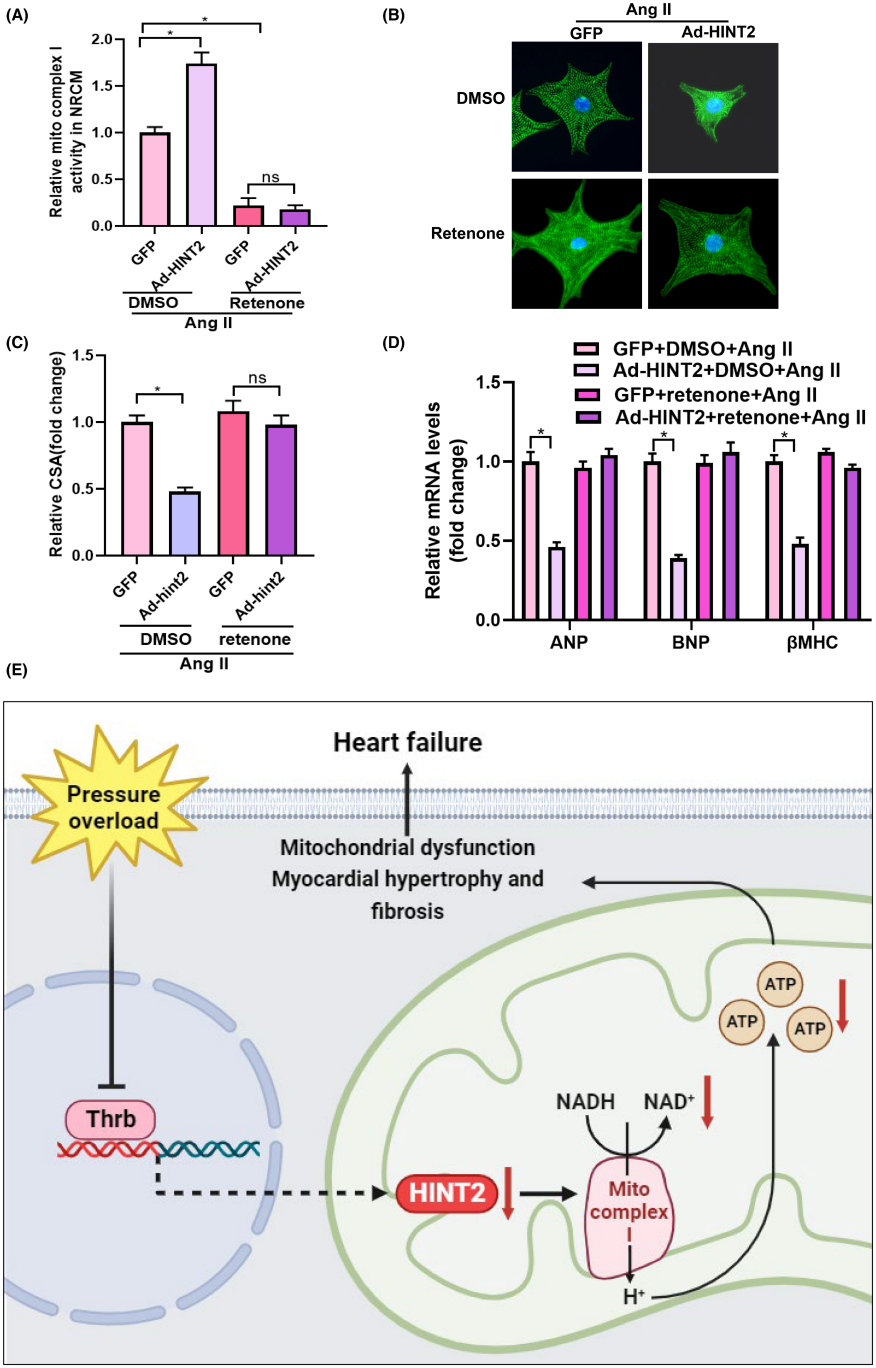
Mitochondrial complex I inhibition offset the protective effect of histidine triad nucleotide‐binding protein 2 (HINT2) in vitro. (A) The activity of mitochondrial complex I (*n* = 6, *p < 0.05). (B, C) α‐actin staining and quantitative results for the detection of the cell surface areas in the indicated groups (*n* > 50 cells per group, *p < 0.05). (D) Transcription of hypertrophic markers in the indicated groups (*n* = 6, *p < 0.05). (E) Schematic images for the regulatory mechanism of HINT2 on pathological cardiac remodelling.

We investigated the involvement of mitochondrial complex I in the cardioprotective effects mediated by HINT2. NRCMs transfected with Ad‐HINT2 were stimulated with Ang II and concurrently treated with rotenone for 48 h. Rotenone effectively attenuated mitochondrial complex I activity (Figure 7A) and reversed the anti‐hypertrophic effects of HINT2 by reducing cell surface area (Figure 7B,C). However, no notable differences were observed in mRNA levels of hypertrophic markers between cells treated with GFP + rotenone + Ang II and Ad‐HINT2 + rotenone + Ang II (Figure 7D). These findings strongly indicate that HINT2's cardioprotective effects primarily involve signalling pathways associated with mitochondrial complex I.

### 3.8 THRB might bind to transcription promoter of HINT2 and regulate its transcription

Finally, we identified the upstream regulator of HINT2. Utilizing FIMO, Jaspar and GeneCards databases, we identified 11 potential transcription factors for HINT2 (Figure S5A). After reviewing literature^27^, ^28^, ^29^ and online sources, we focused on the THRB factor. Correlations between THRB and HINT2 were analysed in GEPIA database samples, indicating a significant correlation (Figure S5B). mRNA expression of THRB and HINT2 was evaluated using GSE198926 microarray dataset, showing a correlation in both TAC and sham mice (Figure S5C). This suggests THRB may regulate HINT2 transcriptionally. Further analysis of HINT2 promoter region using Jaspar database identified potential binding sites for HINT2 and THRB (Figure S5D,E), suggesting THRB as a possible transcription factor for HINT2.

## 4 DISCUSSION

The findings of the current study demonstrated a decrease in HINT2 levels in cardiac remodelling. The overexpression of HINT2 alleviated cardiac function and maintained mitochondrial function following AB. HINT2 enhances the transcription of the NDUF gene, which in turn increases the activity of mitochondrial complex I, oxidative phosphorylation, and ATP synthesis (Figure 7E). Our study findings suggest that HINT2 could potentially serve as a potential therapeutic target for individuals suffering from HF.

Currently, it is known that HINT2 plays a significant role in ischemic heart diseases. Fan et al. discovered that HINT2 expression decreases in failing heart tissues and hypoxic cardiomyocytes, indicating a role in stress response. Overexpression of HINT2 in mice improves post‐myocardial infarction (MI) heart function and preserves MMP and respiration in hypoxic cardiomyocytes.^14^ Li et al. found that HINT2‐deficient mice exhibit increased oxidative stress during myocardial ischemia, which worsens myocardial dysfunction.^15^ Additionally, Li et al. demonstrated that HINT2 interacts with the mitochondrial calcium uniporter, preventing excessive calcium accumulation in mitochondria, mitochondrial fragmentation and apoptosis pathway activation.^11^ These findings suggested that HINT2 plays a crucial role in mitigating the adverse effects of myocardial ischemia on heart function by reducing apoptosis, preserving MMP and enhancing mitochondrial respiration. Our study in pressure‐overload induced HF in mice demonstrated the following: (1) Decreased HINT2 expression in failing hearts, indicating its role in cardiac remodelling and stress response; (2) Protective effects of HINT2, evidenced by improved heart function and reduced damage markers with HINT2 overexpression, and intensified cardiac dysfunction with HINT2 deficiency. We all highlight the significant role of HINT2 in cardiac protection. However, we have proposed a new hypothesis regarding the protective mechanism of HINT2 in the heart. HINT2 may protect against pressure overload‐induced myocardial hypertrophy by regulating the mitochondrial complex I pathway.

Mitochondrial complex I, is the largest complex in the ETC, serving as a redox‐driven proton pump critical for oxidative phosphorylation and aerobic respiration.^30^ The catalytic core of complex I, comprises essential NDUF subunits facilitating electron transfer from NADH to ubiquinone, crucial for oxidative phosphorylation and mitochondrial energy production.^31^, ^32^ Prior research links cardiac hypertrophy pathophysiology to dysfunctional complex I. Mitochondrial dysfunction, particularly involving complex I and II activity, plays a significant role in ventricular hypertrophy, heart failure and cardiac dysfunction. Mitochondrial dysfunction is associated with altered mitochondrial morphology, apoptosis initiation and cardiomyocyte loss, contributing to ventricular decompensation and the development of heart failure.^33^, ^34^, ^35^ In clinical studies, a connection has been established between hypertrophic cardiomyopathy and mutations found in the NDUFS2 and NDUFV2 genes in patients undergoing clinic‐treatment.^36^ In addition, progressive cardiomyopathy development is associated with heteroplasmic alterations in the mitochondrial NADH dehydrogenase 5 gene.^37^ Mutations in the NDUFS6 gene cause severe complex I deficit and neonatal mortality in humans.^38^ A missense variant in the NDUFAF1 subunit is linked to fatal infantile hypertrophic cardiomyopathy due to complex I mis‐assembly.^39^

Many researchers have explored the mechanisms by which mitochondrial complex I regulates myocardial remodelling. Conditional cardiac NDUFS4 deletion mice under excessive cardiac pressure experienced increased cardiac dysfunction and HF compared to WT mice, along with reduced sirtuin 3 activity and NAD+/NADH ratio.^40^ Selective NDUFS4 gene deletion in the heart reduces complex I activity by 50%, leading to severe hypertrophic cardiomyopathy. This disruption of complex I alone is sufficient to cause myocardial dysfunction, independent of ROS‐dependent pathways, highlighting its pivotal role in bioenergetic processes.^41^ Our study findings indicate: (1) HINT2 overexpression led to increased expression and activity of ETC complex I in cardiac mitochondria; (2) HINT2 KO mice showed a reduction in transcriptional and protein levels of NDUF genes associated with mitochondrial complex I, leading to a decline in mitochondrial function.

HINT2 positively regulates the process of oxidative phosphorylation in liver cells and interacts with glucose‐regulated protein 75 (GRP75).^42^ GRP75 binds to cytochrome c oxidase subunit 4, aiding in respiratory chain supercomplex formation. HINT2 deficiency alters mitochondrial protein acetylation, impairing mitochondrial function in mice and affecting respiration and lipid metabolism.^43^ These reports demonstrate the direct targets of HINT2 in regulating mitochondrial function and provided valuable insights. In the future, we will continue researching the downstream targets of HINT2 in the heart. We utilized databases to predict THRB as a potential initiator of the HINT2 transcription program. THRB is widely expressed in cardiac tissues and plays a key role in cardiac disorders.^27^ Reports indicate THRB's regulation of genes linked to myocardial hypertrophy, including Myh6, Adrb1 and Atp2a2,^28^, ^29^ highlighting its role in myocardial remodelling and explaining the downregulation of HINT2 expression. In the future, we plan to conduct experiments to confirm the regulatory effect of THRB on HINT2, further enhancing our understanding of HINT2 and clarifying its upstream and downstream regulatory networks.

Repairing mitochondrial complex I improves cardiac function in HF. Complex I activity is linked to the NAD+/NADH ratio, where NAD+ is the main substrate. Clinical studies show that supplementing NAD+ has a protective effect. For example, nicotinamide riboside, a NAD+ precursor, enhances cardiac function in DCM by increasing NAD+ levels in the failing heart.^44^ Increasing NDUFS1 expression alleviated the diminished activity of complex I and impaired mitochondrial respiratory function post‐MI. Cardiac‐specific NDUFS1 overexpression enhances cardiac function by reducing apoptosis and mitochondrial dysfunction in MI.^45^ These studies suggest that targeting HINT2 may be a viable approach to design therapies aimed at improving mitochondrial function, thereby potentially delaying or even reversing myocardial hypertrophy.

Furthermore, we found that HINT2 enhances AMPKα expression and mitophagy‐related genes, suggesting a potential link between HINT2 and non‐mitochondrial pathways. We will continue to expand this finding in our future work.

To conclude, our study demonstrates that HINT2 preserves heart function and oxidative phosphorylation during pressure overload‐induced cardiac remodelling by maintaining mitochondrial complex I. This suggests that genetics‐based therapy targeting HINT2 could slow the progression of HF by improving the oxidative phosphorylation pathway. Therefore, HINT2 holds promise as a therapeutic agent for cardiac remodelling.

## AUTHOR CONTRIBUTIONS

**Nan Zhang:** Conceptualization (equal); formal analysis (equal); writing – original draft (equal); writing – review and editing (equal). **Zi‐Ying Zhou:** Formal analysis (equal); investigation (lead); methodology (lead); writing – original draft (equal); writing – review and editing (lead). **Yan‐Yan Meng:** Funding acquisition (supporting); project administration (lead); writing – review and editing (supporting). **Hai‐Han Liao:** Conceptualization (equal); data curation (lead); writing – review and editing (supporting). **Shan‐Qi Mou:** Validation (equal); writing – review and editing (supporting). **Zheng Lin:** Validation (equal); writing – review and editing (supporting). **Han Yan:** Investigation (supporting); writing – review and editing (supporting). **Si Chen:** Validation (equal); writing – review and editing (supporting). **Qi‐Zhu Tang:** Funding acquisition (lead); resources (lead); supervision (lead); writing – review and editing (supporting).

## FUNDING INFORMATION

This research was funded by grants from the Regional Innovation and Development Joint Fund of National Natural Science Foundation of China (U22A20269), National Natural Science Foundation of China (82100256) and The Fundamental Research Funds for the Central Universities (2042023kf0045).

## CONFLICT OF INTEREST STATEMENT

The authors declare no conflicts of interest.

## Supporting information


Figure S1.

Table S1.

## Data Availability

The data used to support the findings of this study are available from the corresponding authors upon request.
