# Supplementary material for: HINT2 protects against pressure overload‐induced cardiac remodelling through mitochondrial pathways
Source: J Cell Mol Med. 2024 Mar 28;28(8):e18276. doi: 10.1111/jcmm.18276 (PMC10977391; doi:10.1111/jcmm.18276)
Supplement: Supplementary file 1 — Figure S1. [file JCMM-28-e18276-s001.pdf]

Figure S1

(A)

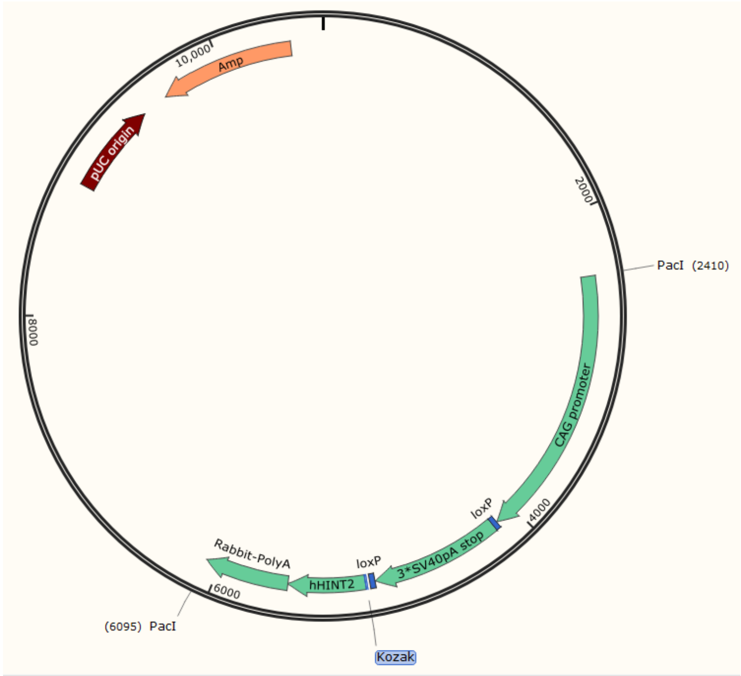

(B)

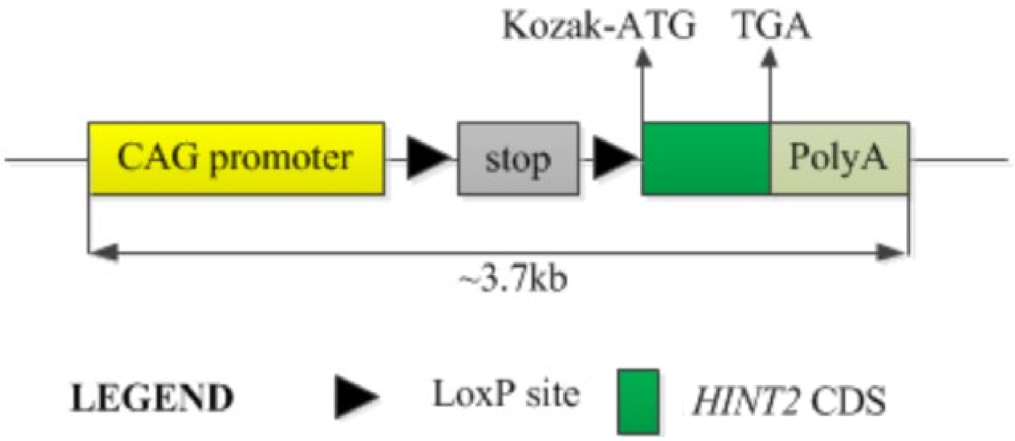

- CAG promoter: chicken beta-actin promoter with CMV enhancer, is a ubiquitous promoter.
- Kozak：Kozak sequence (GCCGCCACC) [1] will be added before the ATG, in order for the ribosome to recognize the initiation codon.

(C)

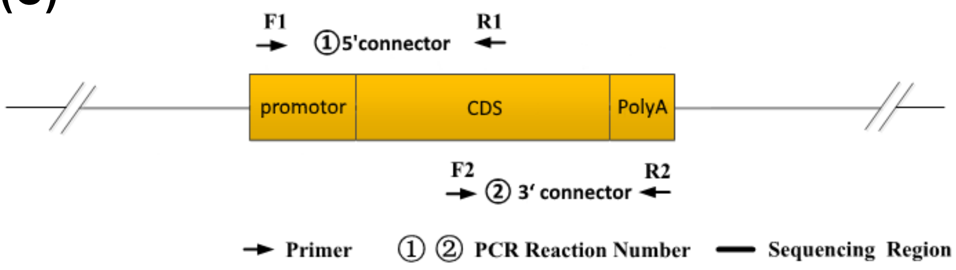

PCR		Sequence
①5'connector	F1	CCGGAACCATAACTTCGTATAGCATA
	R1	CAGCCTGGCTAATCCGAGGAAT
②3'connector	F1	TGAGGACCAGCAGTGTCTTGTGTT
	R1	AGCCTGCACCTGAGGAGTTCAA
③Reference		CTAGGCCACAGAATTGAAAGATCT
		GTAGGTGGAAATTCTAGCATCATCC

(D)

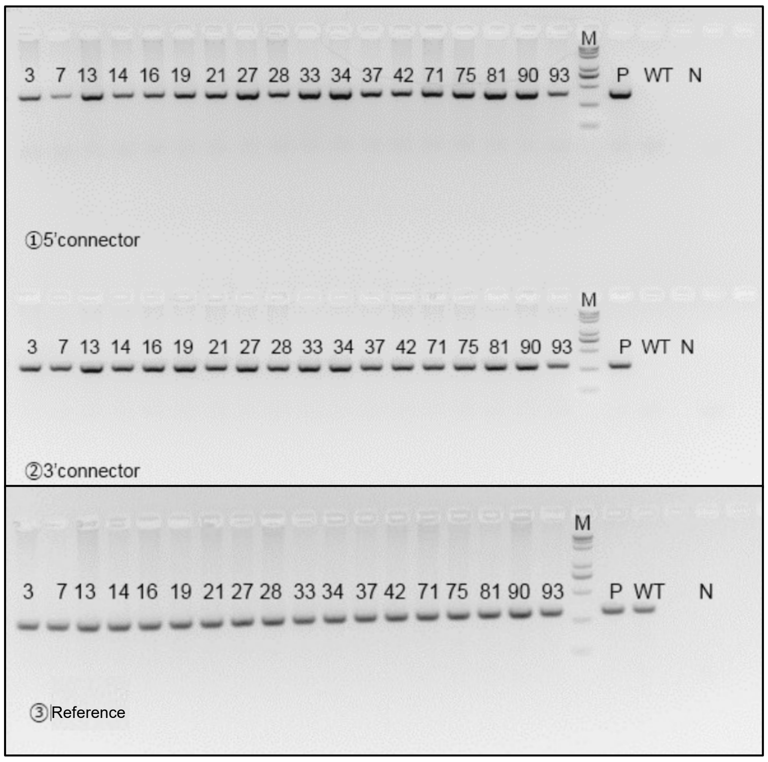

(E)

HINT2$^{fl/fl}$ ✕ αMHC-MerCreMer

↓

HINT2αMHC

↓ Tamoxifem

HINT2-CKO

(F)

Primer	Sequence（5'-3'）
F1	CACCTCTGCCTCTGCCCTCT
R1	TCCCATTCTCCGTCCAAGC

(G)

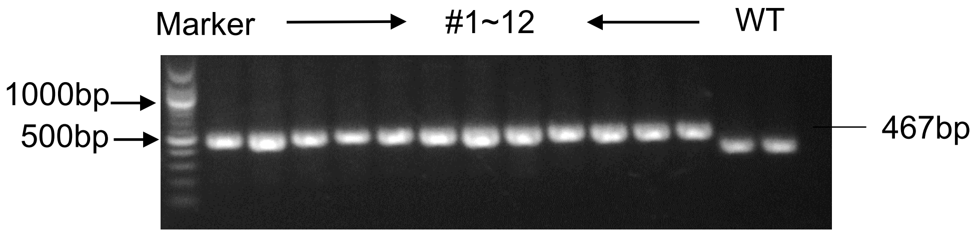

Figure S2

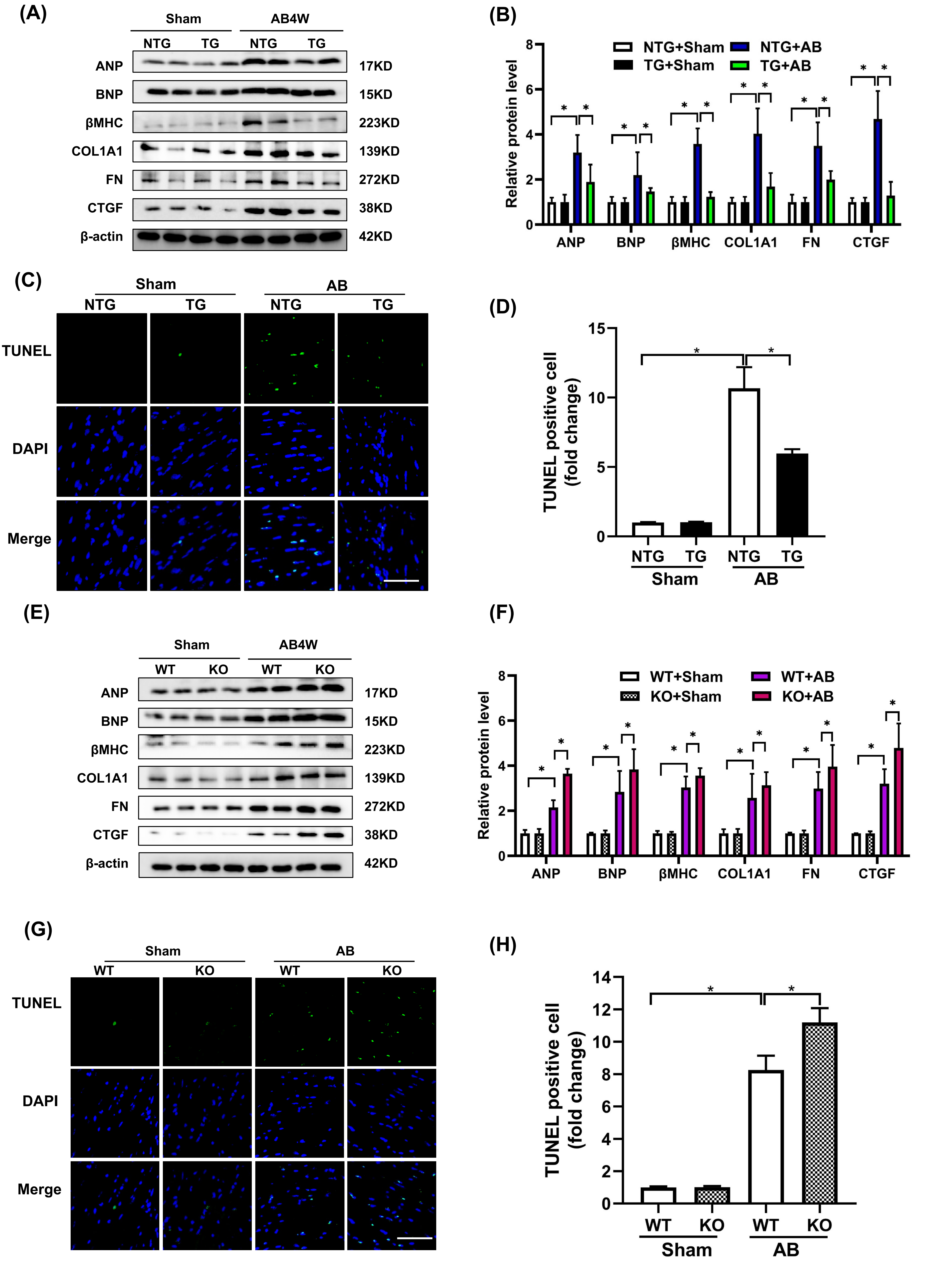

(A)
Sham
AB4W
NTG
TG
ANP
BNP
βMHC
COL1A1
FN
CTGF
β-actin
17KD
15KD
223KD
139KD
272KD
38KD
42KD
(B)
Relative protein level
NTG+Sham
TG+Sham
NTG+AB
TG+AB
(C)
TUNEL
DAPI
Merge
(D)
TUNEL positive cell (fold change)
Sham
AB
(E)
WT
KO
(F)
WT+Sham
KO+Sham
WT+AB
KO+AB
(G)
(H)

Figure S3

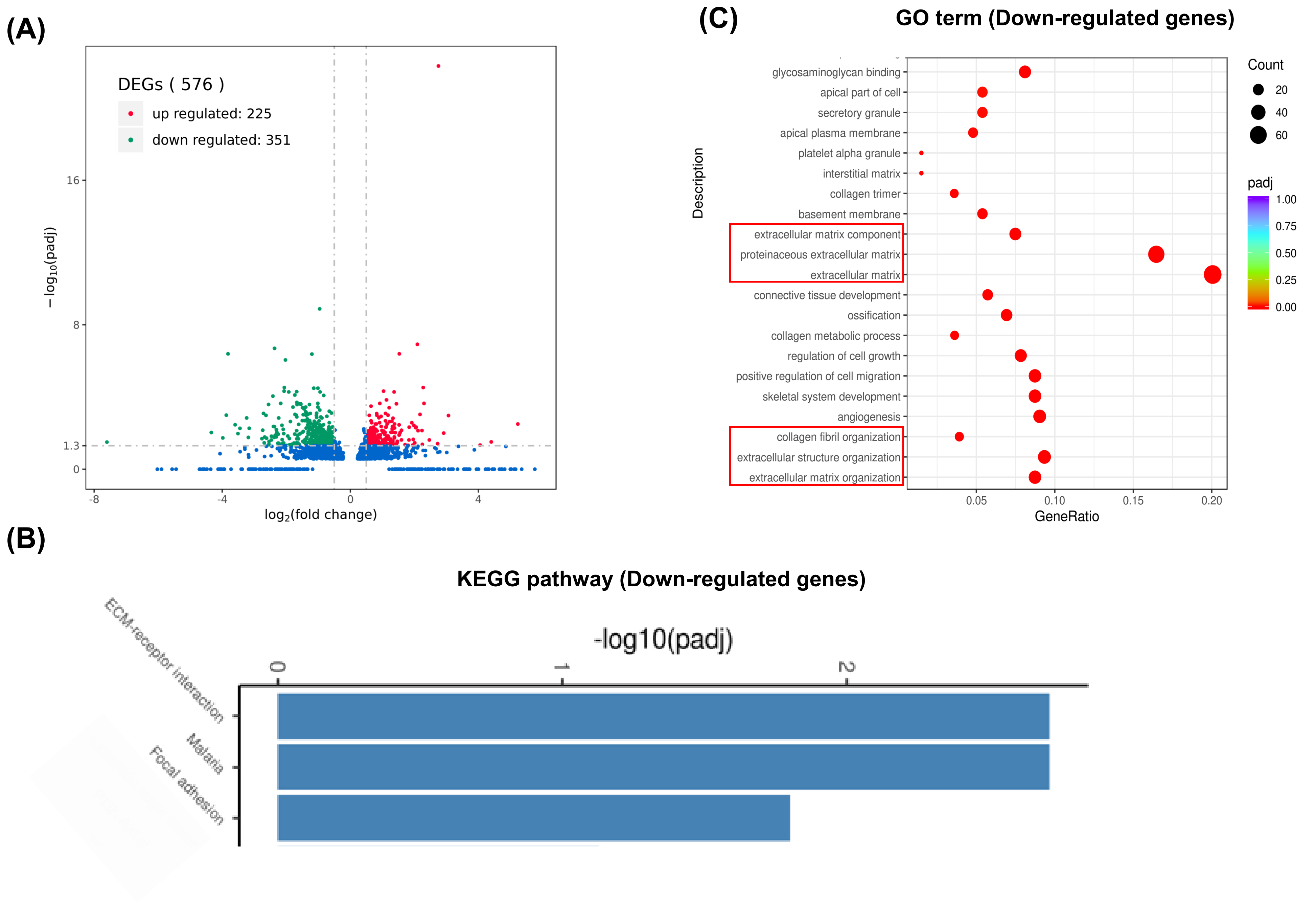
(A)
DEGs (576)
up regulated: 225
down regulated: 351
$-\log_{10}$(padj)
16
8
1.3
0
-8
-4
0
4
$\log_2$(fold change)
(C)
GO term (Down-regulated genes)
Description
glycosaminoglycan binding
apical part of cell
secretory granule
apical plasma membrane
platelet alpha granule
interstitial matrix
collagen trimer
basement membrane
extracellular matrix component
proteinaceous extracellular matrix
extracellular matrix
connective tissue development
ossification
collagen metabolic process
regulation of cell growth
positive regulation of cell migration
skeletal system development
angiogenesis
collagen fibril organization
extracellular structure organization
extracellular matrix organization
0.05
0.10
0.15
0.20
GeneRatio
Count
20
40
60
padj
1.00
0.75
0.50
0.25
0.00
(B)
KEGG pathway (Down-regulated genes)
-log10(padj)
0
1
2
ECM-receptor interaction
Malaria
Focal adhesion

Figure S4

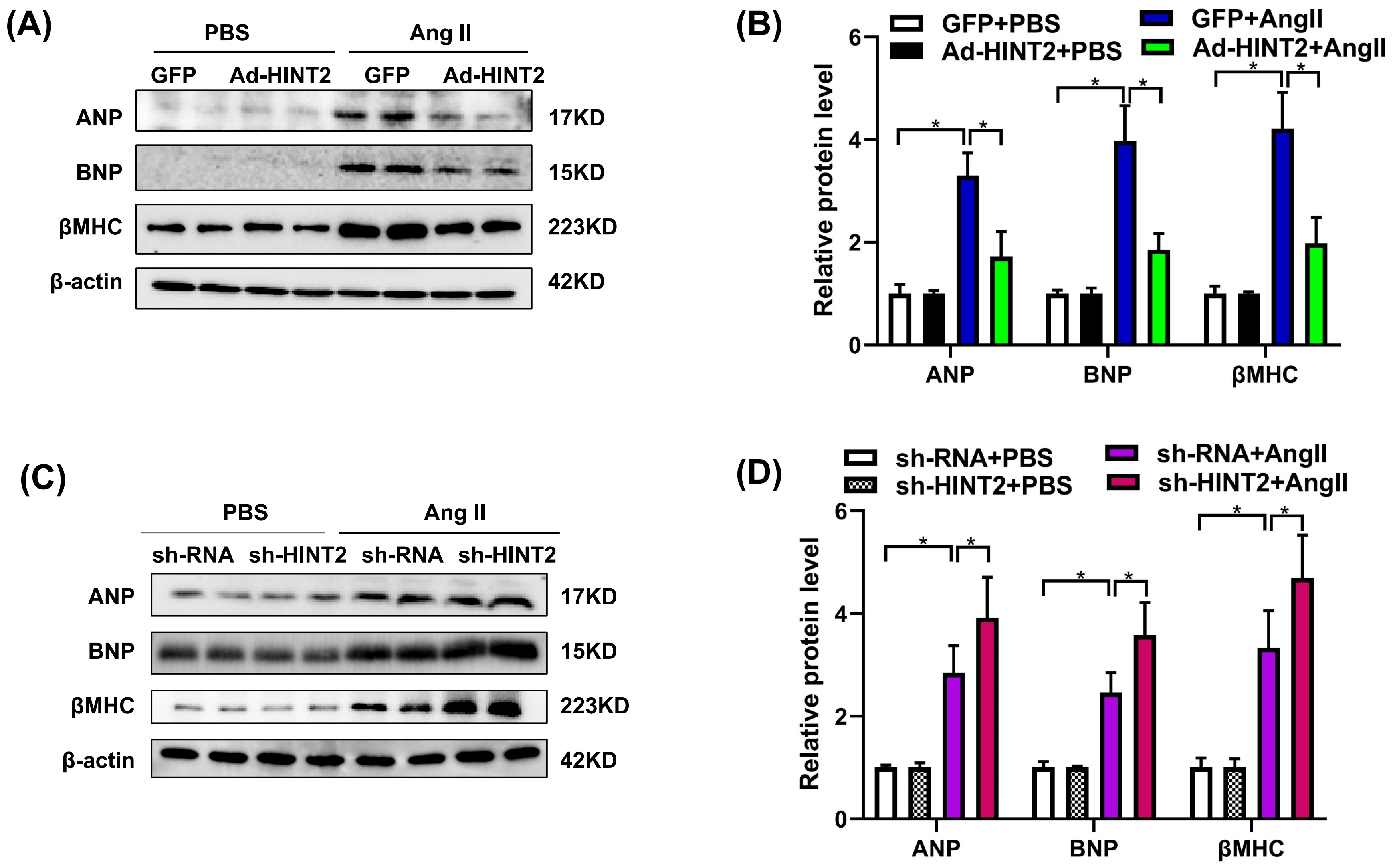

(A)
PBS
Ang II
GFP
Ad-HINT2
GFP
Ad-HINT2
ANP
BNP
βMHC
β-actin
17KD
15KD
223KD
42KD
(B)
GFP+PBS
Ad-HINT2+PBS
GFP+AngII
Ad-HINT2+AngII
Relative protein level
0
2
4
6
ANP
BNP
βMHC
(C)
PBS
Ang II
sh-RNA
sh-HINT2
sh-RNA
sh-HINT2
ANP
BNP
βMHC
β-actin
17KD
15KD
223KD
42KD
(D)
sh-RNA+PBS
sh-HINT2+PBS
sh-RNA+AngII
sh-HINT2+AngII
Relative protein level
0
2
4
6
ANP
BNP
βMHC

Figure S5

(A)

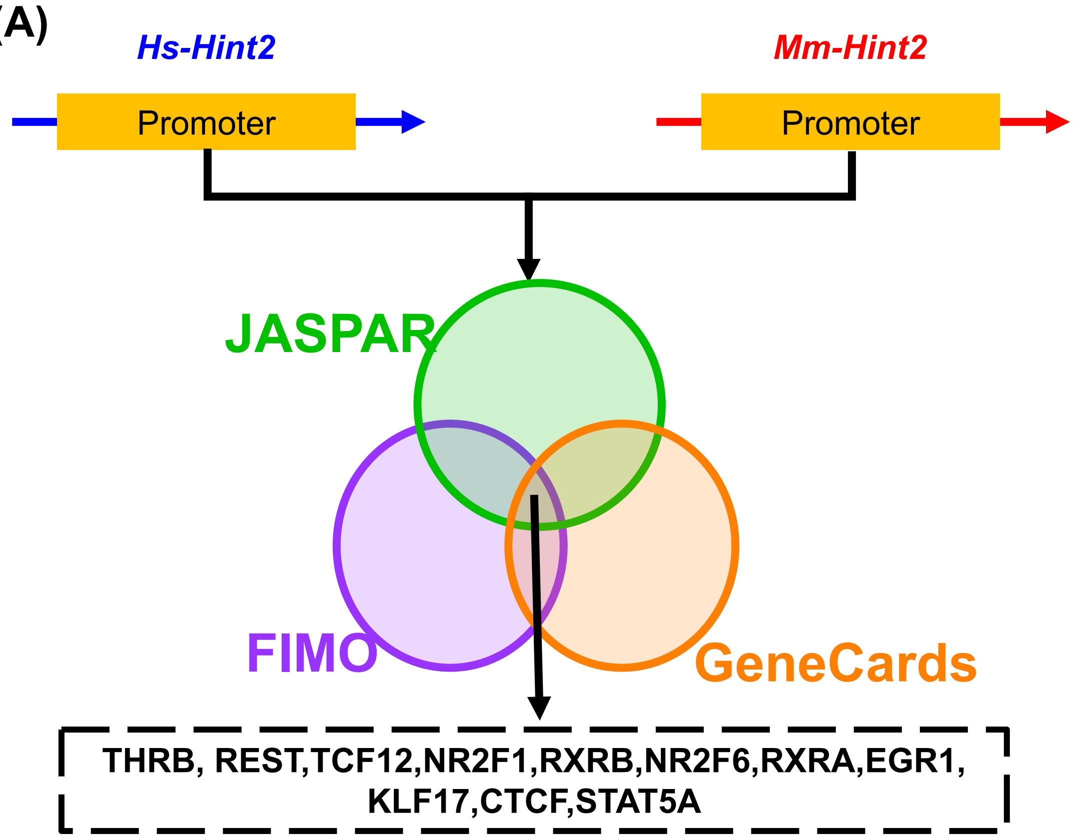

(B)

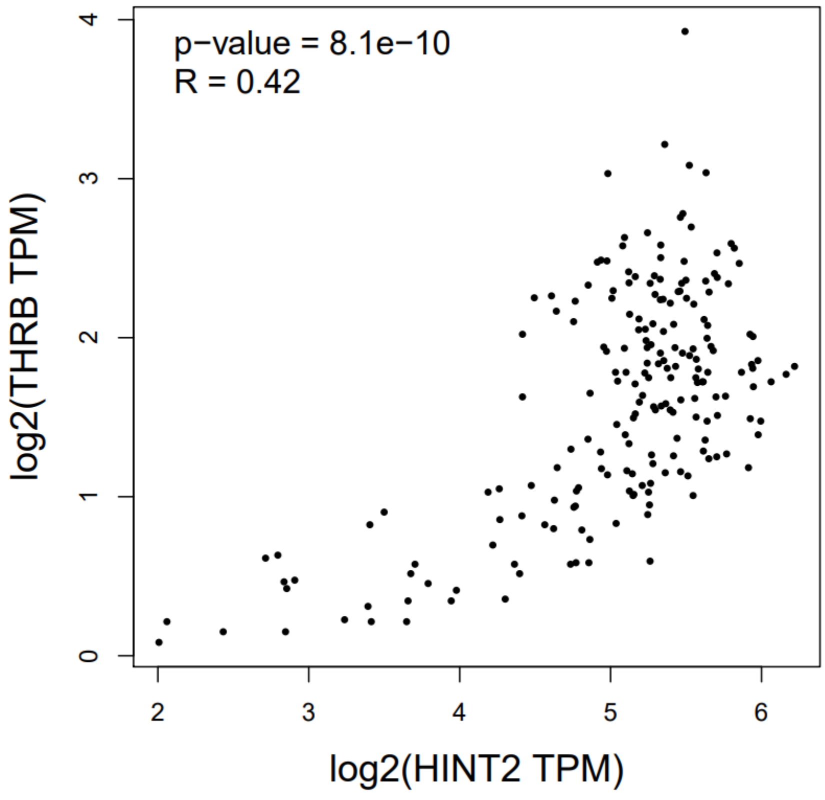

(C)

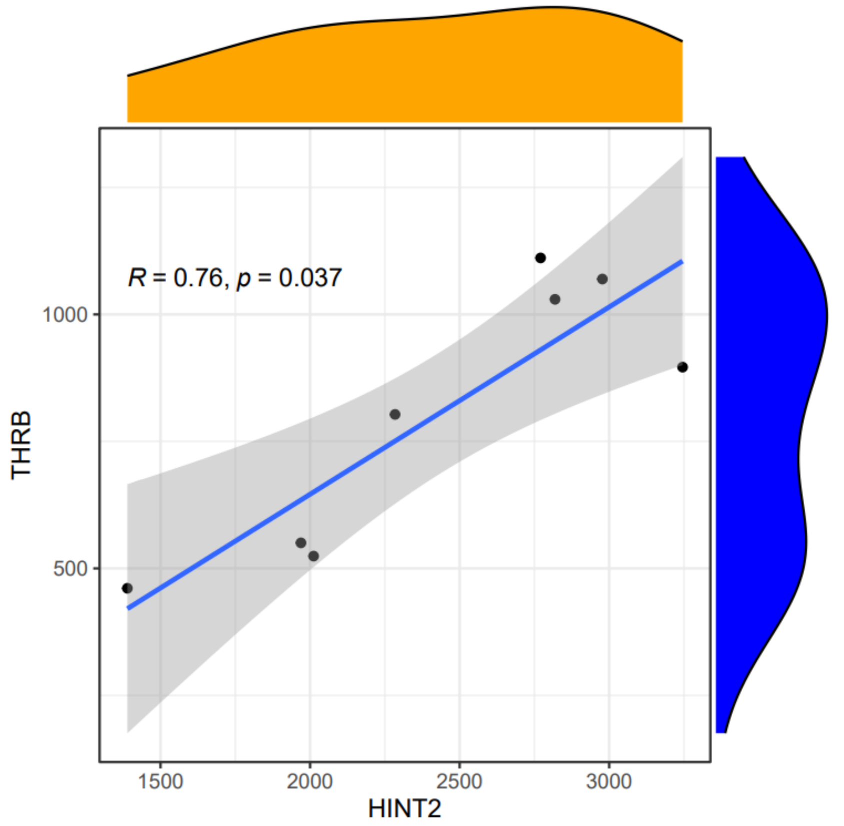

(D)

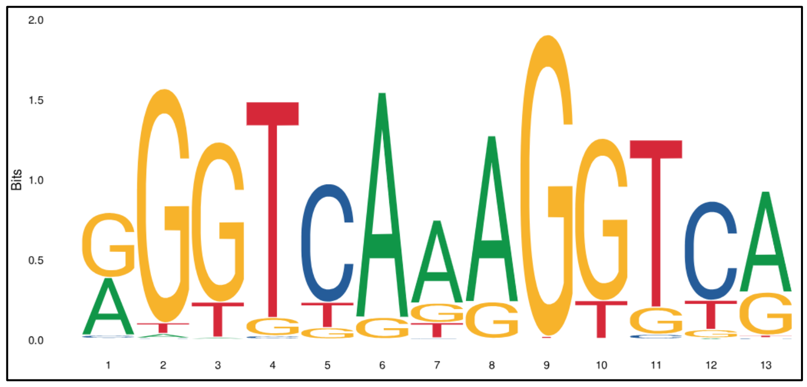

(E)

Human HINT2 promoter:
1 AAAGAAGGCATGAGGATGTGAAGGGAGCATACAGCACCAATAGGGAAAGAAAGTGATGCCAGGAGGCAAA 70
……………………………………………………………………………………………………
771 ATCTCTAGAAGCAAAGTTCCCCAACAGTGGTTATATGCCCCATGA①TAACCATTCACCTAAAACTAATTCA 840
……………………………………………………………………………………………………
1821 AAATGCTGCGCATCAGCCCCGCCAGTTAAAACAAACAAACAAACACAAAAAAACACACCTCATCCCCGT②T 1890
1891 CACCTTTAGCCCCACCCCGGGTGGTTCTTGGAGCCAATAAGCGCCGGATGCCCTCAGAGCCCCGCCCTCC 1960
1961 TCCTGGCCAAAGCGGTGAGAGGGTCTAAAACAGCCCTTTC

Predict sequence 1: AGGTGAATGGTTA
Predict sequence 2:GGGCTAAAGGTGA
