# Supplementary material for: HINT2 protects against pressure overload‐induced cardiac remodelling through mitochondrial pathways
Source: J Cell Mol Med. 2024 Mar 28;28(8):e18276. doi: 10.1111/jcmm.18276 (PMC10977391; doi:10.1111/jcmm.18276)
Supplement: Supplementary file 2 — Table S1. [file JCMM-28-e18276-s002.docx]

**Supplement table 1**

Primer Sequences

| Name | Forward (5’-3’) | Reverse (5’-3’) | |
| --- | --- | --- | --- |
| Mus_HINT2 | TGTTCCGTGACGTAGCTCCT | TCCTAGAAGCTGCTGGTCGT | |
| Mus_β-actin | GCTTCTAGGCGGACTGTTACT | AACCAACTGCTGTCGCCTT | |
| Mus_NPPA | ATTGACAGGATTGGAGCCCAG | TCAAGCAGAATCGACTGCCTT | |
| Mus_NPPB | TTTGGGCTGTAACGCACTGA | CACTTCAAAGGTGGTCCCAGA | |
| Mus_Myh6 | ACATTGGTGCCAAGAAGATGC | GGCAGAGTCGAACGTTTATGT | |
| Mus_Myh7 | CCGAGTCCCAGGTCAACAA | CTTCACGGGCACCCTTGGA | |
| Mus_Col III | TGACTGTCCCACGTAAGCAC | GAGGGCCATAGCTGAACTGA | |
| Mus_Col I | AGCACGTCTGGTTTGGAGAG | GACATTAGGCGCAGGAAGGT | |
| Mus_FN | CCCTGGGTATGACACCGAAA | TTGTCCGCCTAAAGCCATGT | |
| Mus_CTGF | AGACCTGTGCCTGCCATTAC | ACGCCATGTCTCCGTACATC | |
| Mus_Ndufa13 | CTCCCTGAAGTGATGGTGCT | TCCTTTCTTTCGTGAGCCAT | |
| Mus_Ndufb11 | AAATCGCGCTGGGATCAAC | ATGACAGCTTGTACTGCGGA | |
| Mus_Ndufb7 | CTGCTGAAGTGCAAGCGAGA | ATCCAGGTGCTCGCAGTAGT | |
| Mus_Ndufa2 | TTTGGCCAAGAGAAGACGGT | CTTCAGGCTTTGCCGCTTAG | |
| Mus_Ndufa1 | TGCTGCCGGAAGAGAGGTAAA | ACATCTCCGCACCGTTACTC | |
| Mus_Ndufa3 | ATCAACAAGGCCACACCCTA | ATCAGCAGAGGCATTCACAGG | |
| Mus_Ndufs7 | CCGAAAGGTGTACGACCAGA | CGTTGCAGCTGGCACATAGA | |
| Mus_Ndufb8 | TCATGTTCTGGGTAGGGCAC | CCCGCTCCAGGTACAGATTA | |
| Mus_Ndufb6 | TTAAGGCGTACCGCTCCAGT | CCTGGGCTTCGAGCTAACAAT | |
| Mus_Ndufa8 | TTGACGGTCCACACTTGGTC | GGAAAGAACACGAGATCGGC | |
| Rat_HINT2 | ACCACCTGCACATTCACGTA | GGTTCTCCATCCAAGCATCCA | |
| Rat_β-actin | CGCGAGTACAACCTTCTTGCAG | CGCAGCGATATCGTCATCCAT | |
| Rat_NPPA | CGGTACCGAAGATAACAGCCA | TCACCACCTCTCAGTGGCAA | |
| Rat_NPPB | TTTCCTTAATCTGTCGCCGCT | TGCATCGTGGATTGTTCTGGA | |
| Rat_Myh7 | AGTGAAGAGCCTCCAGAGTTTG | | GTTGATGAGGCTGGTGTTCTGG |
| Rat_Ndufa13 | GATGTGCCCAACTGGAAGGT | AACCCATAAAGCTCGCCGAT | |
| Rat_Ndufb7 | AAACGCGACAGCTTTCCCAA | TTCACATAGTCCTGGTGCTCG | |
| Rat_Ndufa2 | TCTGAGTGCTGCTGAGGTGA | CCACATTGTCTCCAGTCCAGT | |
| Rat_Ndufa3 | AGACCAAGATGGCTGCTAGA | TAGTTGTAGGGTGTGGCCTTG | |
| Rat_Ndufs7 | ACCGCTCAGTTTGTGGTTCC | CGGTCCGTCTTCCAAAGAGT | |
| Rat_Ndufa8 | AATTGAAGGAGACGGGCGAC | AGTTGGCAGCTCCACTATCC | |
| H_HINT2 | AGCAGTGTCTTGTGTTCCGT | CCCATCGTTGATCACAAGTCG | |
| H_β-actin | TCACCATGGATGATGATATCGC | GAATCCTTCTGACCCATGCC | |

Mus: Mus musculus; Rat: Rattus; H: Human;
